# Predictors and long-term health outcomes of eating disorders

**DOI:** 10.1371/journal.pone.0181104

**Published:** 2017-07-10

**Authors:** Katie M. O’Brien, Denis R. Whelan, Dale P. Sandler, Janet E. Hall, Clarice R. Weinberg

**Affiliations:** 1 Biostatistics and Computational Biology Branch, National Institute of Environmental Health Sciences, National Institutes of Health, Research Triangle Park, NC, United States of America; 2 Department of Biostatistics and Bioinformatics, Rollins School of Pubic Health, Emory University, Atlanta, GA, United States of America; 3 Epidemiology Branch, National Institute of Environmental Health Sciences, National Institutes of Health, Research Triangle Park, NC, United States of America; 4 Clinical Research Center, National Institute of Environmental Health Sciences, National Institutes of Health, Research Triangle Park, NC, United States of America; Universite de Bretagne Occidentale, FRANCE

## Abstract

Anorexia and bulimia nervosa may have long-term effects on overall and reproductive health. We studied predictors of self-reported eating disorders and associations with later health events. We estimated odds ratios (ORs) for these associations in 47,759 participants from the Sister Study. Two percent (n = 967) of participants reported a history of an eating disorder. Risk factors included being non-Hispanic white, having well-educated parents, recent birth cohort (OR = 2.16, 95% confidence interval [CI]: 2.01–2.32 per decade), and having a sister with an eating disorder (OR = 3.68, CI: 1.92–7.02). As adults, women who had experienced eating disorders were more likely to smoke, to be underweight, to have had depression, to have had a later first birth, to have experienced bleeding or nausea during pregnancy, or to have had a miscarriage or induced abortion. In this descriptive analysis, we identified predictors of and possible long-term health consequences of eating disorders. Eating disorders may have become more common over time. Interventions should focus on prevention and mitigation of long-term adverse health effects.

## Introduction

Eating disorders are relatively rare, with an estimated lifetime prevalence of 0.9% and 1.5%, for anorexia and bulimia nervosa, respectively, among US females [1]. Despite their rarity, eating disorders are life-threatening and also represent a large public health burden due to their potentially long-lasting effects on overall and reproductive health. Other related health outcomes and features of eating disorders include low body mass index (BMI) [1], poor mental health [1–4], substance abuse [3, 4], altered menstrual function [5–7], infertility [8, 9], unplanned pregnancies [8–10], and pregnancy complications such as low birthweight [11–14], gestational diabetes [15], and nausea and vomiting [16].

We used the Sister Study cohort to study the relationships between self-reported eating disorder history and health-related factors. The primary purpose of these descriptive analyses is to assess a range of potential causes and consequences of eating disorders in hopes of informing public health practice and future research endeavors. We were interested in potential predictors of eating disorders, including race, socioeconomic status (SES), birth cohort, and childhood experiences, as well as potential long-term consequences of eating disorders on general health. In a previous analysis in the Sister Study, we found an inverse association between having a history of an eating disorder and incident breast cancer [17]. As many of the known risk factors for breast cancer are related to reproduction and hormonal fluctuations (e.g. age at menarche, age at first birth and other pregnancy characteristics [18, 19]), we were particularly interested in how eating disorders could influence reproductive health. For all analyses, we focused specifically on eating disorders beginning between ages 9–22, as these years encompass key developmental transitions.

## Materials and methods

### Study design

The Sister Study cohort enrolled 50,884 US or Puerto Rican women aged 35–74 who had a sister with breast cancer, but had never been diagnosed with breast cancer (2003–2009). At baseline, participants completed a computer-assisted telephone interview which included questions about demographic and lifestyle characteristics, medical and reproductive history, and environmental exposures. At this time, study examiners took body measurements during an in-home visit. All participants provided written informed consent and the study was approved by Institutional Review Boards at the National Institute of Environmental Health Sciences and Copernicus Group. Requests for data, including the data used in this manuscript, are welcome. De-identified data is made available upon request as described on the Study Website (https://sisterstudy.niehs.nih.gov/English/data-requests.htm). The Sister Study is an ongoing prospective study. The data sharing policy was developed to protect the privacy of study participants and is consistent with study informed consent documents as approved by the NIEHS Institutional Review Board. Dr. Dale Sandler is Principal Investigator of the Sister Study and is responsible for ensuring participant safety and privacy.

Dietary questionnaires were completed separately. These included the question: “Have you ever had anorexia or bulimia?”, to which the respondent could reply “Yes, currently”, “Yes, in the past”, or “No”. Those who answered yes provided values for “How old were you when you first had this?” (0–99) and “For how long did you have this?” (<1 year, 1–2 years, or >2 years). We considered eating disorders that began between ages 9–22, excluding women who did not specify an age (n = 150). We further distinguished between adolescent (ages 9–17) and young-adult (18–22) eating disorders, based on an *a priori* notion that etiology and health consequences could differ by whether onset occurred during the key period of pubertal growth or later in young adulthood.

Women who did not answer the initial eating disorder question but provided an age at eating disorder initiation were included. We made an exception if they also answered yes to the immediately preceding question (n = 30), suspecting that those participants thought they were providing information about onset for that condition instead. We excluded participants who skipped the eating disorder section of the questionnaire (n = 2,294), had missing data for race/ethnicity or childhood SES (n = 550), or who developed breast cancer before completing enrollment or withdrew consent (n = 101). The final sample included 47,759 women.

As the original questionnaire did not ask participants to specify their type of eating disorder or other details, we re-surveyed some women. We successfully re-contacted 581 of 907 women who had self-reported histories of eating disorders and 46 of 99 women who had said they had an eating disorder but had not previously provided an onset age. In the call-back interview, we included the original eating disorder questions plus questions about participants’ eating disorder type (anorexia, bulimia, or both), and questions used to classify the timing and characteristics of the eating disorder (S1 Appendix). All data on eating disorders was self-reported and the questionnaire was not validated. We did not attempt to capture information about eating disorders other than anorexia and bulimia nervosa.

We considered an eating disorder to be confirmed if a participant provided an onset age between 7–24 in the call-back study. Those who said that they did not have an eating disorder, that their eating disorder started outside this range, or who did not respond were set to missing. Among those with confirmed eating disorders, we categorized participants according to whether they had anorexia or bulimia nervosa, with some women included in both categories.

We also identified women with clinically-defined eating disorders, basing the criteria on the Diagnostic and Statistical Manual of Mental Disorders [20]. Among those with confirmed eating disorders, anorexia was considered *clinical* anorexia nervosa if the participant reported at least one of the following: 1) being institutionalized or hospitalized for anorexia; 2) weighing less than 85% of the median for their age (ages 9–19) or BMI ≤17 kg/m^2^ (age ≥20); or 3) having had amenorrhea for 3 months or longer. Bulimia was considered clinically-defined if the participant reported: 1) being institutionalized or hospitalized for bulimia or 2) binge eating with compensatory behavior (e.g. laxative use or vomiting) at least once a week for 3 months.

### Statistical approach

We used dichotomous or polytomous logistic regression to calculate odds ratios (OR) and 95% confidence intervals (CI) for the association between eating disorder status (never versus ever, or never versus either ever with onset ages 9–17 or ever with onset ages 18–22) and the risk factors of interest. Predictors included race/ethnicity, childhood SES (measured by the head of household’s education level when the participant was 13), birth year, childhood food insecurity, and whether the participant had experienced a traumatic life event before age 13. Food insecurity was defined by whether the participant reported that her family had experienced a time when they did not have enough to eat during her childhood. Childhood traumatic events included emotional or physical abuse, serious drug, alcohol or mental health issues in family member(s), unwanted sexual contact, major disasters, accidents or attacks, or the death of a family member or close friend.

Race/ethnicity, childhood SES and birth year were mutually adjusted for and all were included as covariates in the food deprivation and trauma models. When adjusting for birth year as a potential confounder, we generated a restricted cubic spline [21]. To control for within-family dependencies (some participants were siblings) while analyzing a polytomous outcome, we repeatedly created datasets with one randomly selected sister per family, running separate analyses for each of 50 such datasets and averaging results across iterations [22]. For dichotomous outcomes, we used generalized estimating equation (GEE) methods [23]. We used SAS (Cary, NC v9.3) for all analyses.

Possible consequences of eating disorders included parity (0, 1, 2 or ≥3 children), self-reported BMI during ages 30–39 (<18.5, 18.5–24.9, 25–29.9 or ≥30 kg/m^2^), baseline BMI (same categories; based on examiner’s measurements), use of hormonal birth control (ever versus never), cigarette smoking (never smoker, <1, 1–9, or ≥10 pack-years), height (<64, 64–66.9, or ≥67 inches), gynecologic surgical status (no surgery, hysterectomy with retention of ≥1 ovary, or oophorectomy with or without hysterectomy), history of depression (self-reported; includes bipolar disorder), or history of infertility (ever/never sought help for infertility). We again used logistic regression with repeated re-sampling to account for within-family dependencies. Models were adjusted for age at baseline, race/ethnicity, childhood SES, baseline SES (defined by the participant’s education level), and birth year. Women with missing values for baseline SES (n = 4) or the health outcome of interest were excluded.

We also examined the relationship between eating disorder history and select self-reported pregnancy-related outcomes, including preeclampsia, bleeding, nausea with vomiting, gestational hypertension, gestational diabetes, multiple births, miscarriage, low birthweight and preterm birth. We also assessed induced abortion and breastfeeding. We excluded pregnancies in which the participant had taken ovulation-stimulating drugs from the analysis of multiple births and preterm babies from the analysis of low birth weight. Each birth was its own unit of observation and we accounted for multiple pregnancies within women by calculating robust variance estimates using GEE. We adjusted for maternal age at birth, race/ethnicity, childhood SES, baseline SES, and birth year.

In an attempt to capture the association between eating disorders and time-varying factors such as menarche, alcohol and smoking initiation, and sister’s eating disorder status, we also conducted pooled logistic regression analyses using discrete-time Cox models. However, as this information had to be retrospectively reconstructed, the exact event times and their relative temporal ordering may be unreliable (full description in S2 Appendix).

All of the above analyses were repeated to examine possible differences by age at eating disorder onset (9–17 versus 18–22). We also estimated effects for each of the eating disorder types, as defined among those included in the call-back survey (any confirmed disorder, confirmed anorexia nervosa, confirmed bulimia nervosa, clinical anorexia nervosa or clinical bulimia nervosa).

## Results

### Description of cohort

Two percent (n = 967) of participants reported having had an eating disorder that began between age 9 and 22. Compared to women without eating disorders, these women were younger (mean 49.8 years versus 55.8; Table 1) and more likely to be non-Hispanic white (92% versus 85%). They were also more likely to come from a well-educated household, to be well-educated themselves, to be nulliparous, to have had a later first pregnancy, and to have had high physical activity levels as a teen.

**Table 1:** Baseline characteristics of Sister Study participants (2003–2009)^a^.

Characteristic; N (%)	No eating disorder (n = 46,792)	Any history of eating disorder (age 9–22) (n = 967)
**Age at Baseline; mean (SD)**	55.8 (9.0)	49.8 (7.7)
**Age at Menarche; mean (SD)**	12.6 (1.5)	12.7 (1.6)
**Decade of Birth**		
Before 1940	5,661 (12)	24 (2)
1940–1949	15,508 (33)	142 (15)
1950–1959	17,359 (37)	431 (45)
1960 or later	8,264 (18)	370 (38)
**Race/Ethnicity**		
Non-Hispanic White	39,699 (85)	890 (92)
Non-Hispanic Black	3,791 (8)	27 (3)
Hispanic	2,108 (5)	27 (3)
Other	1,194 (3)	23 (2)
**Childhood Socioeconomic Status: Education level of head of household when participant was 13 years old**		
High school or less	25,447 (54)	360 (37)
Some college, associate/ technical degree	8,883 (19)	182 (19)
Bachelor’s degree	7,612 (16)	229 (24)
Master or doctoral degree	4,850 (10)	196 (20)
**Participant Baseline Education Level**		
High school or less	7,063 (15)	64 (7)
Some college, associate/ technical degree	15,763 (34)	269 (28)
Bachelor’s degree	12,671 (27)	334 (35)
Master or doctoral degree	11,292 (24)	299 (31)
Missing	3	1
**Parity**		
0 children	8,498 (18)	240 (25)
1 child	6,747 (14)	158 (16)
2 children	17,256 (37)	325 (34)
≥3 children	14,261 (31)	243 (25)
Missing	30	1
**Age First Term Pregnancy Ended**		
No Full Term Pregnancies	9,441 (20)	264 (27)
<25 y	19,711 (43)	244 (25)
25-<30 y	10,444 (23)	230 (24)
30-<35 y	4,761 (10)	148 (15)
≥35 y	1,894 (4)	77 (8)
Missing	541	4
**Teen Physical Activity (ages 13–19)**		
None	29,288 (63)	465 (49)
1- <5 hours/week	12,463 (27)	313 (33)
5+ hours/week	4,735 (10)	180 (19)
Missing	306	9

^a^50,884 women enrolled in the Sister Study cohort, but we excluded the following from this analysis: women who were missing data for all eating disorder-related questions (n = 2,294); women who provided ambiguous information about eating disorders and age at onset (n = 180); women diagnosed with breast cancer before the completion of the baseline interviews or who withdrew consent (n = 101); women missing data on birth year, childhood SES or race/ethnicity (n = 550).

Eighty percent of women included in the call-back study re-affirmed their eating disorder (n = 462). Thirty women missing age at onset in the original survey provided ages that met the criteria for eating disorders in the call-back study (Table A in S3 Appendix). Among the re-affirmed cases, 276 had anorexia nervosa and 305 had bulimia nervosa (89 with both). Approximately 70% met the clinical criteria (202 for anorexia nervosa and 207 for bulimia nervosa, 41 with both). Baseline characteristics, including age at baseline, age at menarche, birth year, race/ethnicity, child and adult SES, parity, age at first birth and teen physical activity levels, were similar across the two types.

### Predictors of eating disorders

Compared with non-Hispanic whites, Hispanics and non-Hispanic blacks were less likely to report an eating disorder (Table 2). We observed positive associations with childhood SES and birth year, with similar patterns seen for both of the age-at-onset categories and all eating disorder types (Table B in S3 Appendix). Participants who reported a traumatic event or food insecurity during childhood were more likely to report an eating disorder. In supplementary analyses, we found that having a sister with a pre-existing eating disorder was associated with increased risk (OR = 3.68, CI: 1.92–7.02; Table C in S3 Appendix), as was higher childhood/adolescent physical activity (OR = 1.30, CI: 1.14–1.48). For several factors, there was evidence for heterogeneity by age at onset or type of eating disorder. For example, early menarche was associated with an increased risk of developing bulimia nervosa, as was high physical activity and previous alcohol use (Table D1 in S3 Appendix).

**Table 2:** Odds ratios (ORs) and 95% confidence intervals (CIs) for history of eating disorders and selected predictors in the Sister Study (n = 47,759).

	No eating disorder	Eating disorder		Eating disorder, age at onset 9–17 years		Eating disorder, age at onset 18–22 years	
	N (%)	N (%)	OR (95% CI)	N (%)	OR (95% CI)	N (%)	OR (95% CI)
**Race/Ethnicity**^a^							
Non-Hispanic white	39,699 (85)	890 (92)	1.00	464 (91)	1.00	426 (93)	1.00
Non-Hispanic black	3,791 (8)	27 (3)	**0.29 (0.19, 0.42)**	13 (3)	**0.24 (0.14, 0.43)**	14 (3)	**0.33 (0.19, 0.56)**
Hispanic	2,108 (5)	27 (3)	**0.49 (0.33, 0.74)**	17 (3)	**0.47 (0.28, 0.79)**	10 (2)	**0.42 (0.22, 0.80)**
Other	1,194 (3)	23 (2)	**0.78 (0.52, 1.19)**	14 (3)	0.86 (0.49, 1.50)	9 (2)	0.63 (0.32, 1.25)
**Childhood SES**^a^							
High school or less	25,447 (54)	360 (37)	1.00	195 (38)	1.00	165 (36)	1.00
Some college	8,883 (19)	182 (19)	**1.27 (1.06, 1.53)**	101 (20)	1.27 (0.99, 1.63)	81 (18)	1.30 (0.98, 1.71)
Bachelor’s degree	7,612 (16)	229 (24)	**1.67 (1.41, 1.98)**	107 (21)	**1.36 (1.05, 1.74)**	122 (27)	**2.03 (1.59, 2.60)**
Graduate degree	4,850 (10)	196 (20)	**2.13 (1.78, 2.56)**	105 (21)	**1.95 (1.52, 2.51)**	91 (20)	**2.25 (1.71, 2.96)**
**Year of birth**^a^							
<1940	5,661 (12)	24 (3)	1.00	10 (2)	1.00	14 (3)	1.00
1940–49	15,508 (33)	142 (15)	**2.16 (1.40, 3.34)**	74 (15)	**2.62 (1.35, 5.08)**	68 (15)	1.69 (0.94, 3.02)
1950–59	17,359 (37)	431 (44)	**5.81 (3.85, 8.77)**	210 (41)	**6.50 (3.44, 12.3)**	221 (48)	**4.92 (2.85, 8.50)**
≥1960	8,264 (18)	370 (38)	**10.5 (6.93, 15.9)**	214 (42)	**13.7 (7.26, 26.0)**	156 (34)	**7.07 (4.06, 12.3)**
per decade			**2.16 (2.01, 2.32)**		**2.33 (2.08, 2.60)**		**1.94 (1.73, 2.18)**
**Family did not have enough to eat**^b^							
Never	42,495 (91)	880 (91)	1.00	467 (92)	1.00	413 (90)	1.00
Ever	4,276 (9)	87 (9)	**1.30 (1.03, 1.63)**	41 (8)	1.12 (0.80, 1.58)	46 (10)	**1.59 (1.15, 2.19)**
**Traumatic event age<13**^b^^,^^c^							
Never	28,371 (65)	443 (50)	1.00	228 (49)	1.00	215 (51)	1.00
Ever	15,022 (35)	437 (50)	**1.82 (1.59, 2.09)**	234 (51)	**1.82 (1.50, 2.20)**	203 (49)	**1.70 (1.38, 2.08)**

Estimates appear in bold if the 95% confidence interval excludes the null.

^a^Race/ethnicity, head of household’s education level and birth year models are mutually adjusted for each other (with birth year coded as a restricted cubic spline).

^b^Adjusted for race/ethnicity, head of household’s education level, and birth year (as a restricted cubic spline)

^c^3,486 women who did not complete this section of the biennial survey were excluded from this analysis

### Eating disorders and health-related outcomes

There was no clear association between history of an eating disorder and parity (Table 3), but there was a positive association between eating disorder history and subsequently being underweight (BMI <18.5 kg/m^2^) at ages 30–39 or baseline, and an inverse association between eating disorder history and being overweight or obese (BMI ≥25 kg/m^2^). Compared to women without eating disorders, women with eating disorders were more likely to self-report clinically diagnosed depression (OR = 2.17, CI: 1.88–2.49). There was also evidence that women with eating disorders were more likely to have used hormonal birth control and to be moderate-heavy smokers.

**Table 3:** Odds ratios (ORs) and 95% confidence intervals for the association between eating disorder status (Ever ages 9–22, Ever ages 9–18, or Ever ages 18–22) and health-related outcomes (n = 47,759).

	No eating disorder	Eating disorder		Age at onset 9–17		Age at onset 18–22	
	N (%)	N (%)	OR (95% CI)	N (%)	OR (95% CI)	N (%)	OR (95% CI)
**Parity at baseline**							
0 child	8,498 (18)	240 (25)	1.00	108 (21)	1.00	132 (29)	1.00
1 child	6,747 (14)	158 (16)	1.04 (0.84, 1.28)	79 (16)	1.09 (0.80, 1.47)	79 (17)	1.00 (0.75, 1.33)
2 children	17,256 (37)	325 (34)	**0.81 (0.68, 0.97)**	179 (35)	0.96 (0.75, 1.23)	146 (32)	**0.69 (0.54, 0.88)**
≥3 children	14,261 (30)	243 (25)	0.98 (0.81, 1.19)	141 (28)	1.24 (0.95, 1.62)	102 (22)	**0.76 (0.58, 1.00)**
**BMI in 30s**							
Underweight (<18.5 kg/m^2^)	1,194 (3)	46 (5)	**1.80 (1.31, 2.46)**	24 (5)	**1.80 (1.17, 2.77)**	22 (5)	**1.79 (1.15, 2.79)**
Normal (18.5–24.9 kg/m^2^)	35,238 (76)	785 (81)	1.00	405 (80)	1.00	380 (83)	1.00
Overweight (25.0–29.9 kg/m^2^)	7,200 (16)	96 (10)	**0.51 (0.41, 0.64)**	55 (11)	**0.54 (0.40, 0.72)**	41 (9)	**0.48 (0.35, 0.68)**
Obese (≥30.0 kg/m^2^)	2,819 (6)	38 (4)	**0.44 (0.31, 0.62)**	22 (4)	**0.42 (0.26, 0.66)**	16 (3)	**0.47 (0.28, 0.79)**
**Baseline BMI**							
Underweight (<18.5 kg/m^2^)	501 (1)	35 (4)	**2.18 (1.51, 3.15)**	16 (3)	**2.00 (1.18, 3.39)**	19 (4)	**2.37 (1.45, 3.87)**
Normal (18.5–24.9 kg/m^2^)	17,421 (38)	572 (60)	1.00	289 (57)	1.00	283 (62)	1.00
Overweight (25.0–29.9 kg/m^2^)	14,954 (32)	207 (21)	**0.53 (0.45, 0.63)**	113 (22)	**0.56 (0.44, 0.70)**	94 (20)	**0.50 (0.39, 0.64)**
Obese (≥30.0 kg/m^2^)	13,904 (30)	153 (16)	**0.42 (0.35, 0.51)**	90 (18)	**0.48 (0.38, 0.62)**	63 (14)	**0.36 (0.26, 0.48)**
**Use of hormonal birth control**							
Never	6,841 (15)	89 (9)	1.00	50 (10)	1.00	39 (9)	1.00
Ever	39,713 (85)	874 (91)	1.23 (0.98, 1.56)	456 (90)	1.15 (0.84, 1.57)	418 (91)	1.33 (0.94, 1.89)
**Cigarette smoking at baseline**							
Never-smoker	26,360 (57)	533 (56)	1.00	272 (54)	1.00	261 (57)	1.00
<1 pack-year	2,671 (6)	61 (6)	1.12 (0.85, 1.48)	30 (6)	1.12 (0.76, 1.66)	31 (7)	1.12 (0.75, 1.66)
1-<10 pack-years	7,476 (16)	186 (19)	**1.30 (1.10, 1.55)**	93 (18)	1.24 (0.97, 1.58)	93 (20)	**1.37 (1.07, 1.75)**
≥10 pack-years	10,048 (22)	178 (19)	**1.21 (1.01, 1.44)**	108 (21)	**1.46 (1.15, 1.86)**	70 (15)	0.94 (0.71, 1.24)
**Height**							
0–63.9 inches	16,906 (36)	309 (32)	1.00	174 (34)	1.00	135 (29)	1.00
64–66.9 inches	20,439 (44)	436 (45)	0.98 (0.84, 1.14)	234 (46)	0.93 (0.76, 1.14)	202 (44)	1.05 (0.84, 1.32)
≥67 inches	9,446 (20)	222 (23)	0.92 (0.76, 1.10)	100 (20)	**0.72 (0.56, 0.94)**	122 (27)	1.17 (0.90, 1.52)
**Surgical status at baseline**							
None	31,851 (68)	775 (80)	1.00	398 (78)	1.00	377 (82)	1.00
Hysterectomy only	6,447 (14)	74 (8)	0.80 (0.62, 1.03)	35 (7)	0.72 (0.50, 1.04)	39 (8)	0.88 (0.62, 1.24)
Oophorectomy with or without hysterectomy	8,433 (18)	118 (12)	0.93 (0.75, 1.15)	75 (15)	1.25 (0.96, 1.63)	43 (9)	**0.62 (0.44, 0.87)**
**Clinical depression**							
No	37,175 (79)	611 (63)	1.00	312 (61)	1.00	299 (65)	1.00
Yes (includes bipolar)	9,587 (21)	356 (37)	**2.17 (1.88, 2.49)**	196 (39)	**2.36 (1.95, 2.85)**	160 (35)	**1.97 (1.60, 2.41)**
**Ever sought help for infertility**							
No	39,331 (84)	780 (81)	1.00	412 (81)	1.00	368 (80)	1.00
Yes	7,448 (16)	186 (19)	1.12 (0.94, 1.32)	95 (19)	1.08 (0.85, 1.36)	91 (20)	1.16 (0.91, 1.47)

Estimates appear in bold if the 95% confidence interval excludes the null. All models are adjusted for highest education of head of household at age 13, participant’s education level at baseline, age, race/ethnicity, and birth year (as a restricted cubic spline).

There were no overall associations between eating disorders and height, but adolescent-onset (age 9–17) eating disorders (Table 3) and clinical anorexia nervosa (Table E in S3 Appendix) were associated with shorter adult stature. Women with young-adult eating disorders (age 18–22) were less likely to have had an oophorectomy, whereas women with adolescent-onset eating disorders were more likely to have had one. Anorexia nervosa, but not bulimia nervosa, was inversely associated with hysterectomy, and positively associated with infertility.

In reconstructed prospective analyses, young women with eating disorders were less likely to initiate alcohol use by age 22, but more likely to initiate smoking (Table C in S3 Appendix). These associations were stronger for women with a history of anorexia nervosa (Table D2 in S3 Appendix). Women with eating disorders were also more likely to have a sister who subsequently developed an eating disorder and were less likely to give birth than women of the same age who had not developed eating disorders (OR = 0.91, CI: 0.84–0.98). Overall, having a pre-existing eating disorder was not associated with age at menarche, thelarche, or menopause, but women with a history of bulimia nervosa had later menopause.

### Eating disorders and birth-related outcomes

Among parous women (Table 4), history of an eating disorder was associated with a history of bleeding during pregnancy, nausea with vomiting during pregnancy, recognized miscarriage, induced abortion, and preterm birth. We also observed an elevated risk for giving birth to multiples in pregnancies without ovarian hyper-stimulation. Women with a history of eating disorders were more likely to have breastfed (OR = 1.49, CI: 1.25–1.77). We observed no association between eating disorders and any pregnancy-related hypertensive disorders, gestational diabetes, or having a low birth weight term infant. These patterns were generally consistent across eating disorder age-at-onset and type (Table 4 and Table F in S3 Appendix), though the positive association with induced abortions may be specific to those with bulimia nervosa.

**Table 4:** Odds ratios (ORs) and 95% confidence intervals (CIs) for the association between eating disorder status and birth-related outcomes in parous women (n = 38,990).

Outcome (Ever vs. Never)	No eating disorder (115,454 pregnancies to 38,264 women)	Eating disorder (2288 pregnancies to 726 women)		Age at onset 9–17 (1251 pregnancies to 399 women)		Age at onset 18–22 (1037 pregnancies to 327 women)	
	N (%)	N (%)	OR (95% CI)	N (%)	OR (95% CI)	N (%)	OR (95% CI)
**Pre-eclampsia/eclampsia**	3,647 (4)	82 (5)	1.18 (0.92, 1.51)	49 (5)	1.24 (0.90, 1.71)	33 (5)	1.10 (0.75, 1.61)
**Bleeding during pregnancy**	5,352 (6)	134 (8)	**1.37 (1.11, 1.69)**	77 (8)	**1.48 (1.14, 1.91)**	57 (8)	1.23 (0.86, 1.75)
**Nausea with vomiting during pregnancy**	31,056 (34)	578 (35)	**1.25 (1.08, 1.45)**	331 (36)	**1.27 (1.04, 1.54)**	247 (34)	1.22 (0.97, 1.53)
**Pregnancy hypertension**^a^	2,862 (3)	44 (3)	0.78 (0.54, 1.12)	21 (2)	0.67 (0.40, 1.13)	23 (3)	0.92 (0.56, 1.50)
**Gestational diabetes**	3,526 (4)	86 (5)	0.93 (0.70, 1.23)	47 (5)	0.92 (0.64, 1.31)	39 (5)	0.95 (0.61, 1.47)
**Gave birth to multiples**^b^	918 (1)	27 (1)	1.45 (0.99, 2.14)	18 (1)	**1.78 (1.11, 2.86)**	9 (1)	1.06 (0.55, 2.05)
**Miscarriage**	15,488 (13)	396 (17)	**1.19 (1.05, 1.35)**	202 (16)	1.16 (0.99, 1.36)	193 (19)	**1.23 (1.01, 1.51)**
**Induced abortion**	7,415 (6)	222 (10)	**1.25 (1.05, 1.50)**	119 (10)	1.18 (0.92, 1.52)	103 (10)	**1.34 (1.03, 1.74)**
**Breastfed**^c^	54,638 (60)	1,321 (81)	**1.49 (1.25, 1.77)**	730 (81)	**1.45 (1.15, 1.82)**	591 (82)	**1.54 (1.19, 2.00)**
**Low birthweight baby (<5.5 lbs)**^d^	2170 (3)	27 (2)	0.98 (0.65, 1.48)	22 (3)	1.44 (0.90, 2.29)	5 (1)	**0.41 (0.17, 0.99)**
**Preterm birth**^c^	4,960 (6)	113 (7)	1.22 (0.99, 1.51)	65 (7)	1.26 (0.95, 1.68)	48 (7)	1.17 (0.85, 1.61)

Estimates appear in bold if the 95% confidence interval excludes the null. All models are adjusted for highest education of head of household at age 13, participant’s education level at baseline, age, race/ethnicity, and birth year (as a restricted cubic spline)

^a^Excluding births with pre-eclampsia or eclampsia (n = 2,167 births)

^b^Excluding pregnancies where mother took ovulation-stimulating drugs (n = 1,497 pregnancies)

^c^Limited to live births (n = 90,776 in 38,229 women without eating disorders and 1,626 in 725 women with an eating disorder [907 live births in 399 women with eating disorders age 9–17 and 719 live births in 326 women with eating disorders age 18–22]

^d^Limited to live term births (n = 84,844 in 37,060 women without eating disorders and 1,498 in 701 women with an eating disorder [835 live births in 387 women with eating disorders age 9–17 and 664 live births in 314 women with eating disorders age 18–22]

## Discussion

In this descriptive analysis we identified risk factors for and potential consequences of self-reported eating disorders. Risk factors included being non-Hispanic white, higher childhood SES, higher education, younger birth cohort, having experienced food insecurity or a traumatic event during childhood, having a sister with an eating disorder, and higher physical activity levels. Women who had experienced an eating disorder were more likely to start smoking, to be underweight in adulthood, to have used birth control, and to have developed depression. History of an eating disorder was associated with a wide range of adverse pregnancy-related outcomes, including bleeding or nausea with vomiting during pregnancy, miscarriage, induced abortion, and preterm delivery. These findings were generally similar for eating disorders initiated at different ages and of different types, though we observed a few potential differences.

Our observations that white race and high parental education are risk factors for eating disorders are generally consistent with the literature [1, 2, 4, 24, 25]. The relationship between birth cohort and eating disorder incidence is less clear, as rates are difficult to measure and could reflect a heightened awareness of disease rather than true escalations [26, 27]. If the increase is real, it could be attributable to changes in body image idealization, behavioral norms, and the increasing globalization of these characteristics over time. It could also be that younger women are more likely to recall and report this history.

To our knowledge, no previous studies assessed the relationship between childhood food insecurity and eating disorders, but the association between childhood abuse or trauma and eating disorders has been previously reported [28], as has the link between high physical activity and eating disorders [29]. The concordance between eating disorders in sisters observed in our study is compatible with a role of both early environmental and heritable factors in eating disorder etiology. Results from previously conducted genome-wide association studies provide further evidence that genetic factors play an etiologic role [30–32].

Primary and secondary amenorrhea may have health consequences [33]. We found no association between overall eating disorder status and the timing of menarche, but there was some evidence that age at menarche was later in those with pre-existing anorexia nervosa. We did not ask about secondary amenorrhea in the full cohort, but did assess it as part of the criteria for clinical anorexia nervosa in the call-back study.

In accordance with prior studies of eating disorders and mental health [4, 34–37], we found a positive association between eating disorders and depression. Eating disorders were associated with delayed alcohol initiation, but earlier smoking. Both bulimia and anorexia nervosa have been linked to substance abuse disorders [1], but this proclivity may not extend to a calorie-laden substance like alcohol. Cigarette smoking, on the other hand, can suppress appetite [38], and adolescent females often cite weight management as their primary impetus for smoking [39]. (As exploited in the old ad: “Reach for a Lucky instead of a sweet!” [40]).

The negative association between eating disorders and adult BMI was strong and observed for both BMI ages 30–39 and BMI at enrollment. Although limited by sample size, Hudson et al. observed a similar trend among individuals with a history of anorexia nervosa [1]. We also observed an association between adult height and eating disorders that had started during adolescence or that fit the criteria for anorexia nervosa. These findings suggest that eating disorders, especially anorexia nervosa, can have long-lasting impacts on nutrition, body weight, and growth.

The positive relationship between eating disorders and hormonal birth control use may reflect treatment practices, as doctors sometimes prescribe oral contraceptives to regulate menstruation and counteract bone loss in women with anorexia nervosa or amenorrhea [41]. The inverse association between young-adult eating disorders and oophorectomy status is a novel and unexpected finding, the mechanism for which is unclear. A closer investigation of the possible indications for oophorectomy did not elucidate this, as women with eating disorders were more likely to report pelvic pain, endometrial polyps, ovarian cysts, or other gynecologic conditions than were women with no eating disorder history (data not shown). Alternatively, this trend could be related to delayed child-bearing among our highly-educated participants.

Many previous studies of eating disorders and pregnancy-related outcomes were conducted among women who had eating disorders before or during their pregnancies. As these studies were limited to women who eventually became pregnant, they have limited utility for studying fertility, miscarriages, and induced abortions. Nonetheless, several of these studies also reported positive associations between eating disorders and a history of induced abortions, unplanned pregnancies, infertility, or miscarriage [8–10, 15, 42]. The known association between eating disorders and menstrual irregularity [5–7] may explain some of these relationships.

Several studies have also reported that eating disorders during pregnancy are associated with lower birthweights [12–15]. Possible links between eating disorders and pregnancy-related nausea and vomiting [16], twin births [8], pre-eclampsia [43], gestational diabetes [15], and preterm birth [15, 44] are less well-studied. To our knowledge, ours is the first study to consider the relationship between eating disorders and bleeding during pregnancy. We cannot know if these adverse outcomes follow a recurrence of eating disorder symptoms during pregnancy. Other variables, such as BMI and smoking, may also play a role.

There was no difference in breastfeeding initiation by eating disorder status in one prior study [45], but another also observed a positive association [46]. This beneficial outcome of eating disorders may be explained by the belief that breastfeeding can help women reduce weight postpartum [47].

The Sister Study cohort is large, which is necessary to study rare diseases like anorexia and bulimia nervosa. Further, its comprehensive design, which included data spanning birth to present day, allowed us to consider health outcomes over the life course. We were also able to examine the co-occurrence of eating disorders in sisters from the same family.

The Sister Study includes high proportions of well-educated and non-Hispanic white women and all of the participants had at least one sister with breast cancer. While this could limit the generalizability of our results, this sample included a large number of women with eating disorders. As this is a volunteer cohort, there may have been selection bias if women with eating disorders were less likely to participate. This includes possible survivor bias if some women with very severe eating disorders died from their disease. Nevertheless, our estimate of a 2% eating disorder prevalence is similar to the 2.4% prevalence estimated in a recent US population-based survey [1].

Anorexia and bulimia nervosa can co-occur and share some risk factors and health effects, but also have important differences that we were unable to evaluate in the full cohort. Another type of eating disorder, binge eating disorder, was excluded entirely. Our call-back sub-study allowed for some separate investigations, and we were also able to examine the impact of disease severity and look at adolescent and young-adult eating disorders separately. These distinctions across sub-phenotypes provide information about which individuals are at the highest risk of specific health outcomes.

Our use of self-reported eating disorder status is a major limitation. Because they completed the dietary questionnaire on their own, participants may have been more likely to skip or misunderstand the questions. Some women may have been confused about the definition of eating disorders or may have misreported their status because of stigma or social desirability bias. Our findings may not be comparable to other studies that rely on stricter clinical definitions.

On the other hand, this self-reported status likely included individuals with milder eating disorders than would not have been identified if we had recruited from eating disorder clinics or hospital admissions records. Approximately 70% of our participants with eating disorders met the criteria for clinical eating disorders, and Hudson et al. [1] previously reported that half of individuals with eating disorders seek treatment. Therefore, our study may have encompassed a broader representation of the affected subpopulation than would a clinic-based study.

In summary, we identified risk factors for and potential long-term health consequences of eating disorders. We found that women were more likely to have had an eating disorder if they were non-Hispanic white, had a high childhood socioeconomic status, had a sister with an eating disorder, experienced food insecurity or a traumatic event during childhood, or were born more recently. The latter is particularly concerning in terms of public health impact, as it may indicate that incidence has increased over time in the United States.

Possible health consequences of eating disorders that developed during adolescence or early adulthood include altered body size (including low BMI and short stature), increased likelihood of being a smoker or developing depression, and a number of adverse reproductive-related outcomes such as increased likelihood of having a miscarriage or induced abortion, increased risk of delivering preterm, and increased risk of bleeding or nausea and vomiting during pregnancy. This study highlights the need for heightened awareness, especially before and during pregnancy, of women with a history of anorexia or bulimia nervosa. Public health interventions should focus on preventing eating disorders and identifying strategies to mitigate the associated long-term adverse health outcomes.

## Supporting information

S1 Appendix Call-back questionnaire. (DOCX)

S2 Appendix Time-Varying analyses. (DOCX)

S3 Appendix Supplementary tables and figures. (DOCX)

## Abbreviations

BMI: Body Mass Index
CI: Confidence Interval
GEE: Generalized Estimating Equations
HR: Hazard Ratio
OR: Odds Ratio
SES: Socioeconomic status

## References

[1] Hudson JI, Hiripi E, Pope HG Jr., Kessler RC. The prevalence and correlates of eating disorders in the National Comorbidity Survey Replication. Biol Psychiatry. 2007;61(3):348–58. doi: 10.1016/j.biopsych.2006.03.040 ; PubMed Central PMCID: PMCPMC1892232. 16815322 10.1016/j.biopsych.2006.03.040 PMC1892232

[2] Swanson SA, Crow SJ, Le Grange D, Swendsen J, Merikangas KR. Prevalence and correlates of eating disorders in adolescents. Results from the national comorbidity survey replication adolescent supplement. Arch Gen Psychiatry. 2011;68(7):714–23. doi: 10.1001/archgenpsychiatry.2011.22 . 21383252 10.1001/archgenpsychiatry.2011.22 PMC5546800

[3] Hjern A, Lindberg L, Lindblad F. Outcome and prognostic factors for adolescent female in-patients with anorexia nervosa: 9- to 14-year follow-up. Br J Psychiatry. 2006;189:428–32. doi: 10.1192/bjp.bp.105.018820 . 17077433 10.1192/bjp.bp.105.018820

[4] Steinhausen HC, Jakobsen H, Helenius D, Munk-Jorgensen P, Strober M. A nation-wide study of the family aggregation and risk factors in anorexia nervosa over three generations. Int J Eat Disord. 2015;48(1):1–8. doi: 10.1002/eat.22293 . 24777686 10.1002/eat.22293

[5] Misra M, Aggarwal A, Miller KK, Almazan C, Worley M, Soyka LA, et al Effects of anorexia nervosa on clinical, hematologic, biochemical, and bone density parameters in community-dwelling adolescent girls. Pediatrics. 2004;114(6):1574–83. doi: 10.1542/peds.2004-0540 . 15574617 10.1542/peds.2004-0540

[6] Poyastro Pinheiro A, Thornton LM, Plotonicov KH, Tozzi F, Klump KL, Berrettini WH, et al Patterns of menstrual disturbance in eating disorders. Int J Eat Disord. 2007;40(5):424–34. doi: 10.1002/eat.20388 . 17497704 10.1002/eat.20388

[7] Ruuska J, Kaltiala-Heino R, Koivisto A-M, Rantanen Pi. Puberty, sexual development and eating disorders in adolescent outpatients. European Child & Adolescent Psychiatry. 2003;12(5):214–20. doi: 10.1007/s00787-003-0340-4 14667108 10.1007/s00787-003-0340-4

[8] Micali N, dos-Santos-Silva I, De Stavola B, Steenweg-de Graaff J, Jaddoe V, Hofman A, et al Fertility treatment, twin births, and unplanned pregnancies in women with eating disorders: findings from a population-based birth cohort. BJOG. 2014;121(4):408–16. doi: 10.1111/1471-0528.12503 ; PubMed Central PMCID: PMCPMC4155863. 24206173 10.1111/1471-0528.12503 PMC4155863

[9] Easter A, Treasure J, Micali N. Fertility and prenatal attitudes towards pregnancy in women with eating disorders: results from the Avon Longitudinal Study of Parents and Children. BJOG. 2011;118(12):1491–8. doi: 10.1111/j.1471-0528.2011.03077.x . 21810162 10.1111/j.1471-0528.2011.03077.x

[10] Bulik CM, Hoffman ER, Von Holle A, Torgersen L, Stoltenberg C, Reichborn-Kjennerud T. Unplanned pregnancy in women with anorexia nervosa. Obstetrics and Gynecology. 2010;116:1136–40. doi: 10.1097/AOG.0b013e3181f7efdc 20966699 10.1097/AOG.0b013e3181f7efdc PMC3632206

[11] Ekeus C, Lindberg L, Lindblad F, Hjern A. Birth outcomes and pregnancy complications in women with a history of anorexia nervosa. BJOG. 2006;113(8):925–9. doi: 10.1111/j.1471-0528.2006.01012.x . 16827829 10.1111/j.1471-0528.2006.01012.x

[12] Linna MS, Raevuori A, Haukka J, Suvisaari JM, Suokas JT, Gissler M. Pregnancy, obstetric, and perinatal health outcomes in eating disorders. Am J Obstet Gynecol. 2014;211(4):392 e1-8. doi: 10.1016/j.ajog.2014.03.067 . 24705128 10.1016/j.ajog.2014.03.067

[13] Solmi F, Sallis H, Stahl D, Treasure J, Micali N. Low birth weight in the offspring of women with anorexia nervosa. Epidemiol Rev. 2014;36:49–56. doi: 10.1093/epirev/mxt004 ; PubMed Central PMCID: PMCPMC3873840. 24025351 10.1093/epirev/mxt004 PMC3873840

[14] Micali N, Stemann Larsen P, Strandberg-Larsen K, Nybo Andersen AM. Size at birth and preterm birth in women with lifetime eating disorders: a prospective population-based study. BJOG. 2016;123(8):1301–10. doi: 10.1111/1471-0528.13825 . 26697807 10.1111/1471-0528.13825

[15] Micali N, Simonoff E, Treasure J. Risk of major adverse perinatal outcomes in women with eating disorders. Br J Psychiatry. 2007;190:255–9. doi: 10.1192/bjp.bp.106.020768 . 17329747 10.1192/bjp.bp.106.020768

[16] Torgersen L, Von Holle A, Reichborn-Kjennerud T, Berg CK, Hamer R, Sullivan P, et al Nausea and vomiting of pregnancy in women with bulimia nervosa and eating disorders not otherwise specified. Int J Eat Disord. 2008;41(8):722–7. doi: 10.1002/eat.20564 ; PubMed Central PMCID: PMCPMC3247760. 18528877 10.1002/eat.20564 PMC3247760

[17] O'Brien KM, Whelan DR, Sandler DP, Weinberg CR. Eating Disorders and Breast Cancer. Cancer Epidemiology Biomarkers & Prevention. 2016 doi: 10.1158/1055-9965.epi-16-0587 27756775 10.1158/1055-9965.EPI-16-0587 PMC5296273

[18] Kelsey JL, Gammon MD, John EM. Reproductive factors and breast cancer. Epidemiologic Reviews. 1993;15(1):36–47. 8405211 10.1093/oxfordjournals.epirev.a036115

[19] Xue F, Michels KB. Intrauterine factors and risk of breast cancer: a systematic review and meta-analysis of current evidence. The Lancet Oncology. 2007;8(12):1088–100. doi: 10.1016/S1470-2045(07)70377-7 18054879 10.1016/S1470-2045(07)70377-7

[20] American Psychiatric Association. Diagnostic and statistical manual of mental disorders. 5 ed. Washington, DC2013.

[21] Harrell F. Regression Modeling Strategies: with applications to linear models, logistic regression, and survival analysis New York, NY: Springer-Verlag; 2001 6 5, 2001.

[22] Hoffman EB, Sen PK, Weinberg CR. Within-cluster resampling Biometrika. 2001;88(3):1121–34.

[23] Wei LJ, Lin DY, Weissfeld L. Regression analysis of multivariate incomplete failure time data by modeling marginal distributions. Journal of the American Statistical Association 1989;84(408):1065–73.

[24] Alegria M, Woo M, Cao Z, Torres M, Meng XL, Striegel-Moore R. Prevalence and correlates of eating disorders in Latinos in the United States. Int J Eat Disord. 2007;40 Suppl:S15–21. doi: 10.1002/eat.20406 ; PubMed Central PMCID: PMCPMC2680162. 17584870 10.1002/eat.20406 PMC2680162

[25] Franko DL. Race, ethnicity, and eating disorders: Considerations for DSM-V. Int J Eat Disord. 2007;40:S31–S4. doi: 10.1002/eat.20455 17879288 10.1002/eat.20455

[26] Smink FR, van Hoeken D, Hoek HW. Epidemiology of eating disorders: incidence, prevalence and mortality rates. Curr Psychiatry Rep. 2012;14(4):406–14. doi: 10.1007/s11920-012-0282-y ; PubMed Central PMCID: PMCPMC3409365. 22644309 10.1007/s11920-012-0282-y PMC3409365

[27] Lucas AR. The eating disorder "epidemic": More apparent than real? Pediatric Annals. 1992;21(11):746–50. 1484749 10.3928/0090-4481-19921101-09

[28] Mitchison D, Hay PJ. The epidemiology of eating disorders: genetic, environmental, and societal factors. Clin Epidemiol. 2014;6:89–97. doi: 10.2147/CLEP.S40841 ; PubMed Central PMCID: PMCPMC3933432. 24728136 10.2147/CLEP.S40841 PMC3933432

[29] Mendelsohn FA, Warren MP. Anorexia, bulimia, and the female athlete triad: evaluation and management. Endocrinol Metab Clin North Am. 2010;39(1):155–67, x. doi: 10.1016/j.ecl.2009.11.002 . 20122456 10.1016/j.ecl.2009.11.002

[30] Boraska V, Franklin CS, Floyd JA, Thornton LM, Huckins LM, Southam L, et al A genome-wide association study of anorexia nervosa. Mol Psychiatry. 2014;19(10):1085–94. doi: 10.1038/mp.2013.187 ; PubMed Central PMCID: PMCPMC4325090. 24514567 10.1038/mp.2013.187 PMC4325090

[31] Wade TD, Gordon S, Medland S, Bulik CM, Heath AC, Montgomery GW, et al Genetic variants associated with disordered eating. Int J Eat Disord. 2013;46(6):594–608. doi: 10.1002/eat.22133 ; PubMed Central PMCID: PMCPMC3775874. 23568457 10.1002/eat.22133 PMC3775874

[32] Wang K, Zhang H, Bloss CS, Duvvuri V, Kaye W, Schork NJ, et al A genome-wide association study on common SNPs and rare CNVs in anorexia nervosa. Mol Psychiatry. 2011;16(9):949–59. doi: 10.1038/mp.2010.107 ; PubMed Central PMCID: PMCPMC3859494. 21079607 10.1038/mp.2010.107

[33] Warren MPaS, A. L. Exercise and female adolescents: effects on the reproductive and skeletal systems. J Am Med Womens Assoc. 1999;54(3):115–1120. 10441915

[34] Gershon ES, Schreiber JL, Hamovit JR, Dibble ED, Kaye W, Nurnberger JI Jr., et al Clinical findings in patients with anorexia nervosa and affective illness in their relatives. Am J Psychiatry. 1984;141(11):1419–22. doi: 10.1176/ajp.141.11.1419 6496786 10.1176/ajp.141.11.1419

[35] Hudson JI, Pope HG, Jonas JM, Yurgelun-Todd D. Family history study of anorexia nervosa and bulimia. The British Journal of Psychiatry. 1983;142(2):133–8. doi: 10.1192/bjp.142.2.133 6573224 10.1192/bjp.142.2.133

[36] Hudson JI, Pope HG, Jonas JM, Yurgelun-Todd D, Frankenburg FR. A controlled family history study of bulimia. Psychological Medicine. 1987;17:883–90. 3432462 10.1017/s0033291700000684

[37] Strober M, Morrell W, Burroughs J, Salkin B, Jacobs C. A controlled family study of anorexia nervosa. J Psychiatry Research. 1985;19(2/3):239–46. 10.1016/0022-3956(85)90024-x 4045742

[38] Audrain-McGovern J, Benowitz NL. Cigarette smoking, nicotine, and body weight. Clin Pharmacol Ther. 2011;90(1):164–8. doi: 10.1038/clpt.2011.105 ; PubMed Central PMCID: PMCPMC3195407. 21633341 10.1038/clpt.2011.105 PMC3195407

[39] Fulkerson J, French S. Cigarette smoking for weight loss or control among adolescents: gender and racial/ethnic differences. Journal of Adolescent Health. 2003;32(4):306–13. doi: 10.1016/s1054-139x(02)00566-9 12667735 10.1016/s1054-139x(02)00566-9

[40] Stanford School of Medicine. Stanford Research into the Impact of Tobacco Advertising http://tobacco.stanford.edu/tobacco_main/slogans.php: Stanford School of Medicine; [cited 2016 September 22]. Available from: http://tobacco.stanford.edu/tobacco_main/slogans.php.

[41] Haberland CA, Seddick D, Marcus R, Bachrach LK. A physician survey of therapy for exercise-associated amenorrhea: A brief report. Clinical Journal of Sports Medicine. 1995;5(4):246–50. 10.1097/00042752-199510000-00007 7496850

[42] Linna MS, Raevuori A, Haukka J, Suvisaari JM, Suokas JT, Gissler M. Reproductive health outcomes in eating disorders. Int J Eat Disord. 2013;46(8):826–33. doi: 10.1002/eat.22179 . 23996114 10.1002/eat.22179

[43] Micali N, De Stavola B, dos-Santos-Silva I, Steenweg-de Graaff J, Jansen PW, Jaddoe VW, et al Perinatal outcomes and gestational weight gain in women with eating disorders: a population-based cohort study. BJOG. 2012;119(12):1493–502. doi: 10.1111/j.1471-0528.2012.03467.x . 22901019 10.1111/j.1471-0528.2012.03467.x

[44] Micali N, Stemann Larsen P, Strandberg-Larsen K, Nybo Andersen AM. Size at birth and preterm birth in women with lifetime eating disorders: a prospective population-based study. BJOG: An International Journal of Obstetrics & Gynaecology. 2016;123(8):1301–10. doi: 10.1111/1471-0528.13825 26697807 10.1111/1471-0528.13825

[45] Torgersen L, Ystrom E, Haugen M, Meltzer HM, Von Holle A, Berg CK, et al Breastfeeding practice in mothers with eating disorders. Matern Child Nutr. 2010;6(3):243–52. doi: 10.1111/j.1740-8709.2009.00208.x . 20929496 10.1111/j.1740-8709.2009.00208.x PMC6860678

[46] Micali N, Simonoff E, Treasure J. Infant feeding and weight in the first year of life in babies of women with eating disorders. J Pediatr. 2009;154(1):55–60 e1. doi: 10.1016/j.jpeds.2008.07.003 . 18783793 10.1016/j.jpeds.2008.07.003

[47] Baker JL, Gamborg M, Heitmann BL, Lissner L, Sorensen TI, Rasmussen KM. Breastfeeding reduces postpartum weight retention. Am J Clin Nutr. 2008;88(6):1543–51. doi: 10.3945/ajcn.2008.26379 . 19064514 10.3945/ajcn.2008.26379

