# Supplementary material for: Predictors and long-term health outcomes of eating disorders
Source: PLoS One. 2017 Jul 10;12(7):e0181104. doi: 10.1371/journal.pone.0181104 (PMC5507321; doi:10.1371/journal.pone.0181104)
Supplement: S1 Appendix — (DOCX) [file pone.0181104.s001.docx]

**S1 Appendix: Call-back questionnaire given to those with previously reported eating disorders (n=581 of 907 attempted) or possible eating disorders (n=46 of 99 attempted).**

We are contacting you again to get some more detailed information about your history with possible eating disorders.

1. **Have you ever had anorexia or bulimia?**
   1. Yes, anorexia

DE1

- 1. Yes, bulimia
  2. Yes, both
  3. No (GO TO QUESTION 20)

**<ASK IF ANOREXIA OR BOTH>**

DE2

1. **How old were you when your anorexia first developed?** (FILL IN AGE)
2. **Do you currently have anorexia?**

DE3

- 1. Yes (SKIP next question)
  2. No

DE4

1. **How old were you when you last had anorexia?** (FILL IN AGE)

**<ASK IF BULIMIA OR BOTH>**

DE5

1. **How old were you when your bulimia first developed?** (FILL IN AGE)

DE6

1. **Do you currently have bulimia?**
   1. Yes (SKIP next question)
   2. No

DE7

1. **How old were you when you last had bulimia?** (FILL IN AGE)

**<ASK IF YES TO EITHER EATING DISORDER>**

DE8

1. **Sometimes women with eating disorders stop getting their periods. Did this ever happen to you?**
   1. Yes
   2. No (SKIP NEXT QUESTION)
   3. Not applicable – I had not had my first period yet when my eating disorder started (SKIP NEXT QUESTION)

DE9

1. **For how long did you stop getting your period?**
   1. 1 or 2 months
   2. 3 months- 1 year
   3. more than 1 year
2. **When you had an eating disorder, did you experience episodes of binge eating where you ate a large amount of food during a very short period of time?**

DE10

- 1. Yes
  2. No (SKIP NEXT QUESTION)

**11. Approximately how often did you engage in binge eating?**

1. Sporadically/only a few times

DE11

1. Once a week for at least 3 months
2. At least twice a week for at least 3 months
3. Every day or almost every day for at least 3 months

**12. Did you ever engage in any of the following behaviors to control your weight?**

**(CHECK ALL THAT APPLY):**

DE12_01

- 1. Self-induced vomiting

DE12_02

- 1. Use of laxatives, diuretics, enemas or other medications

DE12_03

- 1. Fasting (SKIP TO QUESTION 14)
  2. Extreme exercise (SKIP TO QUESTION 14)

DE12_04

- 1. None (SKIP TO QUESTION 14)

DE12_05

**<ASK IF THEY CHECKED #1 FOR QUESTION 12– SELF-INDUCED VOMITING >**

**13a. Approximately how often did you make yourself vomit?**

DE13A

1.      Sporadically/only a few times

2.      Once a week for at least 3 months

3.      At least twice a week for at least 3 months

4.      Every day or almost every day for at least 3 months

**<ASK IF THEY CHECKED #2 FOR QUESTION 12 – USE OF LAXATIVES, DIURETICS, ENEMAS, OR OTHER MEDICATIONS >**

**13b. Approximately how often did you use laxatives, diuretics, enemas, or other medications to control your weight?**

1.      Sporadically/only a few times

DE13B

2.      Once a week for at least 3 months

3.      At least twice a week for at least 3 months

4.      Every day or almost every day for at least 3 months

1. **Did you ever receive treatment for your eating disorder?**
   1. Yes

DE14

- 1. No (SKIP NEXT QUESTION)

1. **How old were you when you were first treated?**

(FILL IN AGE)

DE15

1. **Were you ever hospitalized or institutionalized for your eating disorder?**
   1. Yes

DE16

- 1. No

1. **What was your lowest weight during the time you had an eating disorder?**

(FILL IN WEIGHT)

DE17

1. **When you were at your lowest weight, how tall were you?**

(FILL IN HEIGHT)

DE18

1. **Did you experience eating disorder symptoms during any of your pregnancies?**
   1. Yes
   2. No
   3. Not applicable - never pregnant

DE19

1. **Has a health care professional ever told you that you are lactose intolerant or allergic to dairy products?**
   1. Yes

DE20

- 1. No

1. **Do you get indigestion or gas after you eat dairy products like milk, yogurt, or cheese?**
   1. Yes

DE21

- 1. No

**<ASK QUESTION 22 IF QUESTIONS 20 OR 21 ANSWERED AS YES>**

1. **At what age did you first notice this lactose sensitivity?**

(FILL IN AGE)

DE22
