# Supplementary material for: Predictors and long-term health outcomes of eating disorders
Source: PLoS One. 2017 Jul 10;12(7):e0181104. doi: 10.1371/journal.pone.0181104 (PMC5507321; doi:10.1371/journal.pone.0181104)
Supplement: S2 Appendix — (DOCX) [file pone.0181104.s002.docx]

**S2 Appendix: Time-varying Analyses**

Time-varying predictors, including menarche, thelarche, alcohol use, smoking status, physical activity, first term pregnancy, and sister’s eating disorder status, were assessed using pooled logistic regression (a discrete-time Cox model). Here, the odds ratios (OR) were estimates of the relative odds of developing an eating disorder for an exposed versus unexposed participant at age t, given that she did not have an eating disorder at age t-1. Exposure status was updated for each age based on information retrospectively reconstructed using the baseline survey. We used generalized estimating equation (GEE) methods to obtain a robust variance estimate that accounted for within-family clustering. All models were adjusted for race/ethnicity, childhood SES, and birth year. When modeling the effects of alcohol and smoking on eating disorder status, we adjusted each for the other.

We then conducted similar pooled logistic, time-to-event analyses looking at the effect of eating disorders on menarche, thelarche, alcohol initiation, smoking initiation and sister’s eating disorder status within the specified time frame (ages 9-22). We also considered whether history of eating disorder affected first occurrence of a term pregnancy (ages 10-48) or onset of natural menopause (age 20-71). As before, these analyses were conducted using pooled logistic regression with GEE and adjustment for race/ethnicity, childhood SES, and birth year. We also adjusted for the participant’s SES at baseline, as measured by her completed education level and models for smoking and alcohol were each adjusted for the other. In the time to natural menopause analysis, women who had a hysterectomy or bilateral oophorectomy were censored at the age their surgery occurred.
