# Supplementary material for: Predictors and long-term health outcomes of eating disorders
Source: PLoS One. 2017 Jul 10;12(7):e0181104. doi: 10.1371/journal.pone.0181104 (PMC5507321; doi:10.1371/journal.pone.0181104)
Supplement: S3 Appendix — (DOCX) [file pone.0181104.s003.docx]

**S3 Appendix: Supplementary Tables and Figures**

**Table A: Baseline characteristics of Sister Study participants with eating disorders by type of disorder (2003-2009)^a^**

| **Characteristic; N (%)** | **Any history of eating disorder (n=967)** | **Confirmed eating disorder (n=492)^a^** | **Anorexia nervosa (n=276)^b^** | **Bulimia nervosa (n=305)^b^** | **Clinical anorexia nervosa (n=202)** | **Clinical bulimia nervosa (n=207)** |
| --- | --- | --- | --- | --- | --- | --- |
| **Age at Baseline; mean (SD)** | 49.8 (7.7) | 49.6 (7.4) | 50.0 (7.5) | 48.8 (7.4) | 50.0 (7.4) | 48.8 (7.0) |
| **Age at Menarche; mean (SD)** | 12.7 (1.6) | 12.8 (1.6) | 12.8 (1.8) | 12.7 (1.5) | 13.0 (1.8) | 12.6 (1.5) |
| **Decade of Birth** |  |  |  |  |  |  |
| Before 1940 | 24 (2) | 10 (2) | 7 (3) | 5 (2) | 6 (3) | 3 (1) |
| 1940-1949 | 142 (15) | 65 (13) | 39 (14) | 35 (11) | 29 (14) | 23 (11) |
| 1950-1959 | 431 (45) | 237 (48) | 136 (49) | 141 (46) | 95 (47) | 96 (46) |
| 1960 or later | 371 (38) | 180 (37) | 94 (34) | 124 (41) | 72 (36) | 85 (41) |
| **Race/Ethnicity** |  |  |  |  |  |  |
| Non-Hispanic White | 890 (92) | 461 (94) | 256 (93) | 284 (93) | 190 (94) | 190 (92) |
| Non-Hispanic Black | 27 (3) | 6 (1) | 4 (1) | 4 (1) | 2 (1) | 3 (1) |
| Hispanic | 27 (3) | 13 (3) | 8 (3) | 11 (4) | 4 (2) | 9 (4) |
| Other | 23 (2) | 12 (2) | 8 (3) | 6 (2) | 6 (3) | 5 (2) |
| **Childhood Socioeconomic Status: Education level of head of household when participant was 13 years old** |  |  |  |  |  |  |
| High school or less | 360 (37) | 187 (38) | 109 (39) | 113 (37) | 80 (40) | 77 (37) |
| Some college, associate/ technical degree | 182 (19) | 79 (16) | 49 (18) | 45 (15) | 33 (16) | 35 (17) |
| Bachelor’s degree | 229 (24) | 120 (24) | 58 (21) | 80 (26) | 43 (21) | 55 (27) |
| Master or doctoral degree | 196 (20) | 106 (22) | 60 (22) | 67 (22) | 46 (23) | 40 (19) |
| **Participant Baseline Education Level** |  |  |  |  |  |  |
| High school or less | 64 (7) | 32 (7) | 19 (7) | 21 (7) | 16 (8) | 17 (8) |
| Some college, associate/ technical degree | 269 (28) | 103 (21) | 64 (23) | 57 (19) | 45 (22) | 45 (22) |
| Bachelor’s degree | 334 (35) | 183 (37) | 98 (36) | 112 (37) | 68 (34) | 73 (35) |
| Master or doctoral degree | 299 (31) | 173 (35) | 94 (34) | 114 (38) | 73 (36) | 71 (34) |
| Missing | 1 | 1 | 1 | 1 | 0 | 1 |
| **Parity** |  |  |  |  |  |  |
| 0 children | 240 (25) | 125 (25) | 68 (25) | 78 (26) | 48 (24) | 57 (28) |
| 1 child | 158 (16) | 80 (16) | 46 (17) | 53 (17) | 33 (16) | 36 (17) |
| 2 children | 325 (34) | 169 (34) | 97 (35) | 101 (33) | 71 (35) | 66 (32) |
| >3 children | 243 (25) | 117 (24) | 65 (24) | 72 (24) | 50 (25) | 47 (23) |
| Missing | 1 | 1 | 0 | 1 |  | 1 |
| **Age First Term Pregnancy Ended** |  |  |  |  |  |  |
| No Full Term Pregnancies | 264 (27) | 136 (28) | 75 (27) | 84 (28) | 53 (26) | 61 (30) |
| <25 y | 244 (25) | 102 (21) | 61 (22) | 61 (20) | 45 (22) | 36 (17) |
| 25-<30 y | 230 (24) | 127 (26) | 69 (25) | 80 (26) | 51 (25) | 59 (29) |
| 30-<35 y | 148 (15) | 82 (17) | 44 (16) | 53 (17) | 36 (18) | 33 (16) |
| >35 y | 77 (8) | 43 (9) | 26 (9) | 25 (8) | 16 (8) | 17 (8) |
| Missing | 4 | 2 | 1 | 2 | 1 | 1 |
| **Teen Physical Activity (ages 13-19)** |  |  |  |  |  |  |
| None | 465 (49) | 235 (48) | 142 (52) | 136 (45) | 101 (50) | 92 (44) |
| 1- <5 hours/week | 313 (33) | 160 (33) | 88 (32) | 104 (35) | 65 (32) | 70 (34) |
| 5+ hours/week | 180 (19) | 92 (19) | 45 (16) | 61 (20) | 35 (17) | 43 (21) |
| Missing | 9 | 5 | 1 | 4 | 1 | 2 |

^a^We re-contacted 907 women who reported eating disorders in the baseline survey, as well as 99 women who said they had a history of an eating disorder but did not provide an age. 627 women (62% response) completed the follow-up survey, including 492 who confirmed that they had an eating disorder (+/- 2 years).

^b^In the follow-up survey, women were asked if they had had 1) anorexia nervosa, 2) bulimia nervosa, 3) both anorexia and bulimia, or 4) no eating disorder. 89 women reported having both and are included in both columns.

^c^Clinical anorexia nervosa defined as: 1) self-reported hospitalization or institutionalization for anorexia; 2) at lowest weight, a weight below the 85^th^ percentile for expected weight for age (ages 9-19) or BMI < 17 kg/m^2^ (age >20); or 3) post-menarche, non-pregnant amenorrhea for 3 months or longer; Clinical bulimia nervosa defined as: 1) binge eating combined with compensatory behavior (e.g. vomiting or laxative use) at least once a week for 3 months; or 2) self-reported hospitalization or institutionalization for bulimia. 41 participants had clinical forms of both disorders and are included in both columns

**Table B. Odds ratios and 95% confidence intervals for type of eating disorders and selected predictors**

|  | **Confirmed eating disorder** | **Anorexia nervosa** | **Bulimia nervosa** | **Clinical anorexia nervosa** | **Clinical bulimia nervosa** |
| --- | --- | --- | --- | --- | --- |
| **Race/Ethnicity^a^** |  |  |  |  |  |
| Non-Hispanic white | 1.00 | 1.00 | 1.00 | 1.00 | 1.00 |
| Non-Hispanic black | 0.12 (0.05, 0.28) | 0.15 (0.05, 0.40) | 0.13 (0.05, 0.35) | 0.10 (0.02, 0.40) | 0.14 (0.05, 0.45) |
| Hispanic | 0.45 (0.25, 0.82) | 0.50 (0.25, 1.03) | 0.60 (0.31, 1.16) | 0.34 (0.13, 0.92) | 0.74 (0.35, 1.55) |
| Other | 0.78 (0.44, 1.39) | 0.94 (0.47, 1.91) | 0.62 (0.28, 1.41) | 0.95 (0.42, 2.15) | 0.78 (0.32, 1.91) |
| **Childhood SES^a^** |  |  |  |  |  |
| High school or less | 1.00 | 1.00 | 1.00 | 1.00 | 1.00 |
| Some college | 1.05 (0.80, 1.37) | 1.13 (0.80, 1.58) | 0.99 (0.70, 1.40) | 1.02 (0.68, 1.54) | 1.14 (0.76, 1.70) |
| Bachelor’s degree | 1.64 (1.29, 2.08) | 1.38 (1.00, 1.91) | 1.79 (1.33, 2.41) | 1.37 (0.94, 2.00) | 1.81 (1.26, 2.59) |
| Graduate degree | 2.15 (1.68, 2.75) | 2.13 (1.55, 2.94) | 2.22 (1.62, 3.03) | 2.18 (1.51, 3.15) | 1.95 (1.32, 2.88) |
| **Year of birth^a^** |  |  |  |  |  |
| <1940 | 1.00 | 1.00 | 1.00 | 1.00 | 1.00 |
| 1940-49 | 2.39 (1.23, 4.65) | 2.05 (0.92, 4.58) | 2.56 (1.00, 6.53) | 1.78 (0.74, 4.29) | 2.80 (0.84, 9.34) |
| 1950-59 | 7.74 (4.11, 14.6) | 6.39 (2.99, 13.6) | 9.10 (3.73, 22.2) | 5.22 (2.29, 11.9) | 10.3 (3.27, 32.6) |
| >1960 | 12.4 (6.57, 23.5) | 9.33 (4.33, 20.1) | 16.8 (6.86, 41.1) | 8.38 (3.65, 19.3) | 19.1 (6.02, 60.7) |
|  |  |  |  |  |  |
| per decade | 2.22 (2.01, 2.44) | 2.09 (1.84, 2.38) | 2.46 (2.16, 2.8) | 2.10 (1.81, 2.44) | 2.51 (2.17, 2.91) |
| **Family did not have enough to eat^b^** |  |  |  |  |  |
| Never | 1.00 | 1.00 | 1.00 | 1.00 | 1.00 |
| Ever | 1.15 (0.82, 1.61) | 1.41 (0.94, 2.1) | 1.31 (0.87, 1.97) | 1.06 (0.62, 1.80) | 1.31 (0.81, 2.11) |
| **Traumatic event age<13^b^** |  |  |  |  |  |
| Never | 1.00 | 1.00 | 1.00 | 1.00 | 1.00 |
| Ever | 2.01 (1.67, 2.41) | 2.08 (1.63, 2.65) | 2.11 (1.68, 2.66) | 1.78 (1.34, 2.37) | 2.02 (1.53, 2.67) |

^a^Race/ethnicity, head of household’s education level and birth year models are mutually adjusted for each other (with birth year coded as a restricted cubic spline).

^b^Adjusted for race/ethnicity, head of household’s education level, and birth year (as a restricted cubic spline)

**Table C. Odds Ratios (ORs) and 95% confidence intervals (CIs) for the association between incident eating disorder status (ages 9-22) and select factors (n=47,759).**

| **Outcome** | **Number of Events^a^** | **Predictor (ages 9-22)** | **Number exposed^b^** | **OR (95% CI)**^c^ |
| --- | --- | --- | --- | --- |
| Eating disorder status (ages 9-22) | 967 | Menarche | 47,665 | 1.32 (0.86, 2.02) |
|  | 951 | Thelarche | 46,604 | 1.09 (0.72, 1.65) |
|  | 958 | Physical activity^d^ | 28,785 | 1.30 (1.14, 1.48) |
|  | 966 | Alcohol use | 41,656 | 1.13 (0.95, 1.34)^e^ |
|  | 966 | Smoking status | 18,669 | 1.09 (0.93, 1.28)^e^ |
|  | 963 | First term pregnancy | 13,933 | 0.63 (0.45, 0.89) |
|  | 207 | Eating disorder in sister^f^ | 177 | 3.68 (1.92, 7.02) |
| Menarche (ages 9-22) | 46,124 | Eating disorder status | 77 | 0.86 (0.60, 1.21) |
| Thelarche (ages 9-22) | 46,286 |  | 79 | 0.99 (0.72, 1.36) |
| Alcohol initiation (ages 9-22) | 41,798 |  | 498 | 0.85 (0.74, 0.97)^e^ |
| Smoking initiation (ages 9-22) | 18,799 |  | 779 | 1.15 (1.00, 1.34)^e^ |
| First term pregnancy (ages 10-48)^g^ | 37,508 |  | 941 | 0.91 (0.84, 0.98) |
| Eating disorder in sister (ages 9-22)^f^ | 160 |  | 198 | 4.10 (1.71, 9.25) |
| Menopause (ages 20-71)^g^ | 28,489 |  | 967 | 1.04 (0.94, 1.15) |

^a^Number of events among participants with non-missing data for the specific predictor/outcome combination.

^b^Prior to experiencing the event or being censored

^c^All models adjusted for race/ethnicity, head of household’s education level at age 13, and birth year (as a restricted cubic spline). For models where eating disorder status is the predictor, we additionally adjusted for participant’s baseline education level.

^d^Physical activity levels (in MET hours/week), comparing women with greater than the median for the age group (0 for childhood, 2 for adolescent) to women who had less than the median for the age group

^e^Smoking and alcohol are mutually adjusted for one another

^f^Analysis limited to 4,366 families with at least 2 included sisters (n=9,222 participants)

^g^Right and left truncated for specified age range to ensure model convergence.

**Table D1. Odds Ratios and 95% confidence intervals for the association between select factors and incident eating disorder type.**

| **Predictor** | **Confirmed eating disorder** | **Anorexia nervosa** | **Bulimia nervosa** | **Clinical anorexia nervosa** | **Clinical bulimia nervosa** |
| --- | --- | --- | --- | --- | --- |
| Menarche | 1.85 (0.98, 3.47) | 1.47 (0.70, 3.07) | 2.76 (1.03, 7.35) | 1.04 (0.48, 2.28) | 2.98 (0.83, 10.7) |
| Thelarche | 1.35 (0.70, 2.58) | 1.61 (0.69, 3.75) | 1.70 (0.65, 4.42) | 1.24 (0.49, 3.08) | 1.87 (0.53, 6.53) |
| Physical activity | 1.28 (1.06, 1.54) | 1.16 (0.90, 1.48) | 1.45 (1.14, 1.85) | 1.22 (0.91, 1.63) | 1.48 (1.10, 1.99) |
| Alcohol use | 1.29 (1.03, 1.61) | 1.03 (0.77, 1.38) | 1.77 (1.32, 2.38) | 0.96 (0.68, 1.36) | 1.54 (1.08, 2.19) |
| Smoking status | 1.07 (0.86, 1.33) | 0.96 (0.71, 1.31) | 1.24 (0.95, 1.62) | 0.99 (0.69, 1.41) | 1.23 (0.89, 1.70) |
| First term pregnancy | 0.35 (0.18, 0.68) | 0.37 (0.15, 0.92) | 0.37 (0.16, 0.83) | 0.43 (0.16, 1.19) | 0.33 (0.12, 0.91) |

**Table D2. Odds Ratios and 95% confidence intervals for the association between incident eating disorder type and select factors.**

| **Outcome** | **Confirmed eating disorder** | **Anorexia nervosa** | **Bulimia nervosa** | **Clinical anorexia nervosa** | **Clinical bulimia nervosa** |
| --- | --- | --- | --- | --- | --- |
| Menarche (ages 9-22) | 0.83 (0.48, 1.44) | 0.68 (0.37, 1.27) | 1.64 (0.78, 3.46) | 0.59 (0.30, 1.15) | 2.27 (0.69, 7.42) |
| Thelarche (ages 9-22) | 0.92 (0.54, 1.57) | 0.92 (0.42, 2.02) | 0.93 (0.49, 1.77) | 0.80 (0.36, 1.78) | 0.67 (0.30, 1.52) |
| Alcohol initiation (ages 9-22) | 0.94 (0.79, 1.12) | 0.74 (0.60, 0.92) | 1.20 (0.92, 1.56) | 0.66 (0.53, 0.82) | 1.09 (0.79, 1.50) |
| Smoking initiation (ages 9-22) | 1.15 (0.95, 1.40) | 1.25 (0.97, 1.60) | 1.14 (0.88, 1.48) | 1.14 (0.85, 1.53) | 1.15 (0.83, 1.60) |
| First term pregnancy (ages 10-48) | 0.91 (0.82, 1.01) | 0.90 (0.78, 1.04) | 0.95 (0.83, 1.08) | 0.91 (0.77, 1.08) | 0.86 (0.73, 1.01) |
| Menopause (ages 20-71) | 0.70 (0.52, 0.95) | 0.98 (0.67, 1.44) | 0.65 (0.43, 0.97) | 0.98 (0.63, 1.52) | 0.71 (0.45, 1.11) |

**Table E. Odds Ratios and 95% confidence intervals for the association between eating disorder type and health-related outcomes**

|  | **Confirmed eating disorder** | **Anorexia nervosa** | **Bulimia nervosa** | **Clinical anorexia nervosa** | **Clinical bulimia nervosa** |
| --- | --- | --- | --- | --- | --- |
| **Parity at baseline** |  |  |  |  |  |
| 0 child | 1.00 | 1.00 | 1.00 | 1.00 | 1.00 |
| 1 child | 1.10 (0.82, 1.47) | 1.10 (0.75, 1.62) | 1.22 (0.85, 1.76) | 1.10 (0.70, 1.74) | 1.08 (0.70, 1.67) |
| 2 children | 0.86 (0.68, 1.10) | 0.87 (0.63, 1.20) | 0.86 (0.63, 1.19) | 0.91 (0.62, 1.33) | 0.74 (0.51, 1.08) |
| >3 children | 0.98 (0.75, 1.29) | 0.92 (0.64, 1.32) | 1.04 (0.74, 1.48) | 1.00 (0.66, 1.53) | 0.87 (0.57, 1.31) |
| **BMI in 30s** |  |  |  |  |  |
| Underweight (<18.5 kg/m^2^) | 2.13 (1.42, 3.19) | 2.80 (1.75, 4.50) | 1.44 (0.78, 2.67) | 2.99 (1.77, 5.06) | 2.80 (1.75, 4.50) |
| Normal (18.5-24.9 kg/m^2^) | 1.00 | 1.00 | 1.00 | 1.00 | 1.00 |
| Overweight (25.0-29.9 kg/m^2^) | 0.52 (0.38, 0.71) | 0.33 (0.20, 0.54) | 0.71 (0.50, 1.00) | 0.21 (0.10, 0.42) | 0.57 (0.36, 0.90) |
| Obese (>30.0 kg/m^2^) | 0.25 (0.13, 0.48) | 0.40 (0.20, 0.80) | 0.29 (0.14, 0.61) | 0.29 (0.11, 0.72) | 0.38 (0.17, 0.85) |
| **Baseline BMI** |  |  |  |  |  |
| Underweight (<18.5 kg/m^2^) | 2.55 (1.61, 4.04) | 3.52 (2.08, 5.94) | 2.16 (1.15, 4.03) | 3.80 (2.14, 6.76) | 2.23 (1.07, 4.65) |
| Normal (18.5-24.9 kg/m^2^) | 1.00 | 1.00 | 1.00 | 1.00 | 1.00 |
| Overweight (25.0-29.9 kg/m^2^) | 0.49 (0.39, 0.62) | 0.41 (0.30, 0.58) | 0.52 (0.39, 0.71) | 0.38 (0.25, 0.55) | 0.44 (0.31, 0.65) |
| Obese (>30.0 kg/m^2^) | 0.33 (0.25, 0.45) | 0.34 (0.23, 0.50) | 0.37 (0.25, 0.53) | 0.24 (0.15, 0.40) | 0.36 (0.24, 0.56) |
| **Use of hormonal birth control** |  |  |  |  |  |
| Never | 1.00 | 1.00 | 1.00 | 1.00 | 1.00 |
| Ever | 1.13 (0.82, 1.55) | 1.14 (0.75, 1.74) | 1.06 (0.72, 1.56) | 1.16 (0.71, 1.92) | 1.18 (0.72, 1.94) |
| **Cigarette smoking at baseline** |  |  |  |  |  |
| Never-smoker | 1.00 | 1.00 | 1.00 | 1.00 | 1.00 |
| <1 pack-year | 1.39 (0.97, 1.99) | 1.39 (0.88, 2.21) | 1.58 (1.02, 2.46) | 1.20 (0.69, 2.11) | 1.48 (0.86, 2.54) |
| 1-<10 pack-years | 1.36 (1.07, 1.73) | 1.10 (0.78, 1.54) | 1.70 (1.27, 2.28) | 1.15 (0.78, 1.68) | 1.53 (1.06, 2.20) |
| ≥10 pack-years | 1.08 (0.83, 1.41) | 1.13 (0.80, 1.58) | 1.12 (0.79, 1.59) | 1.05 (0.70, 1.57) | 1.12 (0.74, 1.70) |
| **Height** |  |  |  |  |  |
| 0-63.9 inches | 1.00 | 1.00 | 1.00 | 1.00 | 1.00 |
| 64-66.9 inches | 1.12 (0.90, 1.38) | 0.93 (0.70, 1.23) | 1.33 (1.01, 1.76) | 0.72 (0.52, 0.99) | 1.36 (0.96, 1.92) |
| >67 inches | 0.94 (0.72, 1.22) | 0.77 (0.54, 1.08) | 1.09 (0.78, 1.51) | 0.64 (0.43, 0.95) | 1.18 (0.79, 1.77) |
| **Surgical status at baseline** |  |  |  |  |  |
| None | 1.00 | 1.00 | 1.00 | 1.00 | 1.00 |
| Hysterectomy only | 0.63 (0.42, 0.94) | 0.44 (0.24, 0.80) | 0.71 (0.43, 1.18) | 0.28 (0.12, 0.66) | 0.70 (0.38, 1.29) |
| Oophorectomy with or without hysterectomy | 1.15 (0.87, 1.51) | 1.10 (0.76, 1.57) | 1.29 (0.91, 1.82) | 1.03 (0.67, 1.57) | 1.41 (0.94, 2.11) |
| **Clinical depression** |  |  |  |  |  |
| No | 1.00 | 1.00 | 1.00 | 1.00 | 1.00 |
| Yes (includes bipolar) | 2.29 (1.88, 2.77) | 2.88 (2.24, 3.71) | 2.03 (1.59, 2.61) | 2.82 (2.10, 3.77) | 2.2 (1.63, 2.95) |
| **Ever sought help for infertility** |  |  |  |  |  |
| No | 1.00 | 1.00 | 1.00 | 1.00 | 1.00 |
| Yes | 1.13 (0.90, 1.43) | 1.42 (1.06, 1.90) | 0.99 (0.73, 1.35) | 1.38 (0.98, 1.95) | 0.98 (0.67, 1.43) |

All models are adjusted for highest education of head of household at age 13, participant’s education level at baseline, age, race/ethnicity, and birth year (as a restricted cubic spline).

**Table F. Odds ratios and 95% confidence intervals for the association between eating disorder status and birth-related outcomes in parous women**

| **Outcome (Ever vs. Never)** | **Confirmed eating disorder** | **Anorexia nervosa** | **Bulimia nervosa** | **Clinical anorexia nervosa** | **Clinical bulimia nervosa** |
| --- | --- | --- | --- | --- | --- |
| **Pre-eclampsia/eclampsia** | 1.23 (0.88, 1.72) | 1.30 (0.85, 2.00) | 1.23 (0.81, 1.88) | 1.25 (0.77, 2.03) | 1.20 (0.72, 1.99) |
| **Bleeding during pregnancy** | 1.38 (1.03, 1.84) | 1.43 (0.97, 2.09) | 1.39 (0.99, 1.97) | 1.42 (0.95, 2.10) | 1.48 (0.98, 2.21) |
| **Nausea with vomiting during pregnancy** | 1.34 (1.09, 1.65) | 1.18 (0.89, 1.55) | 1.40 (1.07, 1.84) | 1.04 (0.75, 1.45) | 1.27 (0.92, 1.77) |
| **Pregnancy hypertension^a^** | 0.86 (0.52, 1.43) | 0.83 (0.43, 1.61) | 1.12 (0.62, 2.03) | 0.69 (0.28, 1.70) | 1.14 (0.54, 2.40) |
| **Gestational diabetes** | 1.05 (0.73, 1.51) | 1.15 (0.74, 1.80) | 0.93 (0.55, 1.57) | 1.14 (0.67, 1.92) | 0.86 (0.44, 1.68) |
| **Gave birth to multiples^b^** | 1.30 (0.74, 2.30) | 0.82 (0.31, 2.19) | 1.72 (0.93, 3.19) | 1.09 (0.41, 2.91) | 1.04 (0.39, 2.81) |
| **Miscarriage** | 1.18 (0.99, 1.41) | 1.10 (0.87, 1.39) | 1.17 (0.93, 1.48) | 1.18 (0.91, 1.54) | 1.26 (0.96, 1.66) |
| **Induced abortion** | 1.16 (0.91, 1.46) | 0.88 (0.64, 1.22) | 1.30 (0.97, 1.74) | 0.82 (0.56, 1.20) | 1.12 (0.77, 1.64) |
| **Breastfed^c^** | 1.83 (1.40, 2.40) | 1.92 (1.35, 2.73) | 1.85 (1.31, 2.61) | 1.95 (1.27, 2.99) | 2.00 (1.29, 3.08) |
| **Low birthweight baby (<5.5 lbs)^d^** | 0.60 (0.30, 1.22) | 0.66 (0.27, 1.60) | 0.37 (0.12, 1.14) | 0.73 (0.27, 1.97) | 0.18 (0.03, 1.26) |
| **Preterm birth^c^** | 1.10 (0.81, 1.51) | 1.10 (0.72, 1.68) | 1.23 (0.85, 1.78) | 1.11 (0.69, 1.80) | 1.31 (0.85, 2.03) |

^a^Excluding births with pre-eclampsia or eclampsia

^b^Excluding pregnancies where mother took ovulation-stimulating drugs

^c^Limited to live births

^d^Limited to live term births
